# Dual intimate partner violence among women in sub-Saharan Africa: The Case of Zambia and Zimbabwe

**DOI:** 10.4102/phcfm.v17i1.4921

**Published:** 2025-09-30

**Authors:** Keatlegile M.E. Mabena, Karabo Mhele, Wandile F. Tsabedze

**Affiliations:** 1 Department of Development Studies, College of Human and Social Sciences, University of South Africa, Pretoria, South Africa; 2 Department of Population Studies and Demography, Faculty of Human and Social Sciences, North-West University, Mahikeng, South Africa; 3 Department of Psychology, College of Human and Social Sciences, University of South Africa, Pretoria, South Africa

**Keywords:** intimate partner violence, women, Zambia, Zimbabwe, sexual violence

## Abstract

**Background:**

Previous research explored the risk factors associated with intimate partner violence (IPV) in the sub-Saharan region, there is a notable paucity of studies addressing cases in which an individual reported experiencing multiple IPV incidents.

**Aim:**

This study aimed to examine the prevalence and sociodemographic factors of dual IPV among women in Zambia and Zimbabwe.

**Setting:**

Zimbabwe and Zambia in 2015 and 2018, respectively.

**Methods:**

Data for this study were obtained from demographic and health surveys. The study included 11 779 (weighted) women aged 15 to 54 years who were selected for questions on domestic violence. Multinomial regression was used to estimate the relative risk of experiencing physical abuse, emotional abuse or both types of violence compared to experiencing none.

**Results:**

While 12% and 9.7% of the respondents reported experiencing only physical and emotional IPV, respectively, almost a quarter (21.7%) were subject to both forms of IPV in the same period. The probability of experiencing both forms of IPV was highest among those whose partners showed controlling behaviour, consumed alcohol, had lower levels of education and had been employed in the past year. The risk of experiencing IPV increased with lower educational attainment among participants, longer relationship duration, number of co-wives, and was higher among those employed.

**Conclusion:**

The study recommends counselling interventions, improved access to education and coordinated responses involving key stakeholders. Addressing IPV requires context-specific strategies, the establishment of safe houses and enhanced data systems to monitor its prevalence and trends.

**Contribution:**

The study emphasises on the mental healthcare for women who experience IPV.

## Introduction

Intimate partner violence (IPV) is a major health risk that mainly affects women in different regions of the world. The World Health Organization (WHO) reports that slightly less than one third (31%) of women aged 15 years and older have experienced violence at some point in their lives.^1^ The problem appears to be significantly worse in sub-Saharan Africa (SSA), where prevalence rates are above the world average, ranging from 36% to 45%.^2,3^

Although other forms of IPV such as sexual violence, economic abuse and psychological manipulation have been reported in the literature, physical and emotional violence are more prevalent and thus require greater attention compared to other forms of violence.^4,5^ Furthermore, although previous research has explored the risk factors associated with IPV in the sub-Saharan region, there is a notable paucity of studies addressing cases in which an individual reported experiencing multiple IPV incidents. This approach enables the measurement of the magnitude of physical and emotional forms of IPV, as well as the identification of key predictors. This approach can provide a deeper understanding of the factors driving the different forms of IPV, and the findings can be valuable in shaping policies and programmes to prevent gender-based violence in these countries.

Demographic and Health Surveys (DHS) have been gathering data from women on the various forms of IPV, which include physical, emotional and sexual violence, primarily in countries across SSA. The findings reveal that IPV is most prevalent in this region.^2,6^ However, disparities exist among different countries, with physical and emotional forms of violence reported most frequently by women.^6,7^ Unfortunately, victims of these violent acts were less likely to report them when they occurred.^8^ This non-reporting behaviour has undermined campaigns aimed at addressing such actions. Factors most associated with IPV across various countries include partner alcohol consumption, controlling partner behaviour, endorsement of wife-beating, lower levels of education and living in lower-income households.^2,6,7,9^ Interestingly, residing in communities that highly accept wife-beating increases the likelihood of abuse for employed women.^10^

Previous studies have shown that Zambia and Zimbabwe have comparatively higher levels of IPV among countries in Southern Africa with a prevalence of more than 40%,^1,2^ making them suitable for this type of study. IPV has increased in Zimbabwe from 40% to 43% in 2010 and 2015 respectively.^11^ Therefore, the focus of the study was on these two countries. Both countries have higher rates of justification for partner violence among men.^12^ Furthermore, more than half of the women in Zambia support partner violence, and in Zimbabwe, a substantial proportion of women also justify such violence under certain conditions, reflecting widespread acceptance of IPV in both contexts.^13^ The acceptance of such behaviour is usually associated with higher rates of IPV. Research indicates significant variations in IPV across SSA. Notably, Middle Africa reports the highest rate of IPV at 49.3%, while West Africa has the lowest at 34.3%.^14^ A separate analysis highlights that physical violence is the most prevalent form of IPV, with Rwanda reporting 49% of cases in 2010, in stark contrast to Nigeria reporting 9% in 2013.^15^ Alcohol use by partners has been identified as a significant contributor to the likelihood of reporting IPV across various countries. This connection is particularly pronounced among women with lower socioeconomic status, indicating that other factors may mitigate the impact of alcohol consumption.^16,17^ Furthermore, the cultural justification of violence correlates with higher instances of IPV; the acceptance of violent behaviour within certain cultural frameworks underscores the importance of addressing this issue.^17^ Research shows that the justification of violence among women ranges between 33% and 57% in various sub-Saharan countries.^17^ Interestingly, a different study links the endorsement of such behaviours to lower levels of education.^18^

Recent findings reinforce the critical role of women’s economic empowerment in reducing IPV. Evidence suggests that higher education levels for both women and men, along with increased household wealth, are directly associated with a significant decrease in the prevalence of IPV.^16,19^ A study conducted in the United States (US) revealed that households experiencing significant economic hardship show a notable increase in the likelihood of individuals engaging in abusive behaviour towards others.^20^ This association underscores the complex interplay between socioeconomic conditions and interpersonal dynamics, suggesting that financial stressors may exacerbate tensions within families and communities, potentially leading to aggression or violence. The economic instability may exacerbate abuse, as victims often struggle to leave their abusers because of financial dependence.^21^

Furthermore, studies have shown that significant increases in the unemployment rate are associated with an increase in controlling behaviour among men towards their romantic partners.^20^ This controlling behaviour is, in turn, positively linked to a higher risk of IPV.^22^

Physical and emotional violence has been associated with different negative effects on the individual and families. In some cases, victims were reported to have developed suicidal ideation.^14,15,23^ Furthermore, victims may develop risky behaviours, such as engaging in unprotected sexual activity and participating in transactional sex, which can increase the risk of contracting human immunodeficiency virus (HIV), among others.^16^ An individual experiencing different types of IPV will be expected to experience more adverse effects than someone who has experienced only one type of IPV.^24^ Physical and emotional violence has far-reaching consequences for the health and functioning of individuals and households. It is linked to a range of negative outcomes, including mental health disorders such as depression and anxiety, physical injuries, chronic illnesses and harmful coping behaviours.^16,17^ These impacts often disrupt relationships and can extend to children, increasing their vulnerability to emotional, behavioural and developmental challenges, and perpetuating cycles of trauma across generations.^2,3^ Early identification and intervention are crucial to preventing further harm, supporting recovery and restoring safety and dignity.^13^ Effectively addressing violence not only improves the well-being of those directly affected but also alleviates pressure on healthcare systems, strengthens social support structures and contributes to building healthier, more resilient communities.^12^ There is a need of evidence-based interventions that can address IPV, such as healthcare-based, community and educational interventions, legal and policy interventions, economic empowerment interventions, perpetrator intervention programmes and technology-based interventions, which are diverse and span individual, community, healthcare and policy levels in an African context.^1,25,26,27,28,29^

Connell’s Gender and Power Framework highlights how social norms, gender-based division of labour and power imbalances shape social relationships and individual experiences in society.^30^ This framework can provide information on the social dynamics through which IPV manifests itself in different societies. Social norms supporting IPV have been documented in countries in SSA where the incidence of IPV is relatively high.^31,32^ These supportive norms create an environment for IPV to thrive, as perpetrators often take advantage (of not being reported to police) of the normalisation of such violence. In particular, the presence of deeply ingrained patriarchal beliefs within society results in the unequal distribution of power based on gender, placing women in a subordinate position to men.^33^ This power dynamic increases men’s inclination to exert control over women, consequently elevating the likelihood of IPV.^3^ Specifically, in the context of SSA, a notable proportion of men exhibit behaviours (controlling and lifetime physical, sexual and emotional violence) geared towards exerting dominance over women. These behaviours have been consistently linked to higher rates of IPV.^3^

In addition, partner violence is often facilitated by the unequal distribution of work based on gender and the resulting economic disparities between the genders. Socioeconomic difficulties, such as widespread poverty and limited access to resources among women, create a conducive environment as victims of such violence become economically dependent on perpetrators, making it difficult to challenge abuse.

## Research methods and design

### Study design

This is a cross-sectional quantitative study using secondary data from the DHS conducted in Zimbabwe and Zambia. The latest surveys were carried out in 2023–2024 in both countries; however, the authors had access to data for Zimbabwe and Zambia only for 2015 and 2018, respectively. The data were, until recently, freely accessible to researchers upon request to the DHS. The data are collected from a representative sample of women in the reproductive age groups (15–49 years). The topics primarily cover a wide range of demographic and health issues, with IPV being one of them. Information on IPV is collected from a sub-sample of the larger group of ever-partnered women. Information about the types of violence a woman experienced from an intimate partner in the past 12 months is collected during the interview. Only one woman per household is randomly selected among those eligible for the interview. To adjust for non-representation, the sample was weighted using the d005 weight to ensure that the data reflects the targeted population. After excluding those not eligible, the total weighted sample in this study was 11 779, of which 6 439 (54.7%) was from Zambia, which is large enough for undertaking the study of this nature.

### Sampling

From the total sample of 16 726 households in the two countries randomly selected to identify eligible participants for the study, 11 779 were eligible for inclusion in the sample. The largest proportion of respondents came from Zambia, contributing 54.7%, while the remaining 45.3% were from Zimbabwe. For this study, we formally requested and obtained permission from the DHS to use and analyse the data. The second sampling stage included randomly selecting households in the selected enumeration area(s) (EAs), and study participants were selected from each of the selected households. Demographic and Health Surveys collected larger sample sizes to detect statistically significant effects and aims at a power of 80%. Further details on sample determination can be obtained on this website: https://www.dhsprogram.com/pubs/pdf/DHSM4/DHS6_Sampling_Manual_Sept2012_DHSM4.pdf.^34^

### Statistical analysis

Data were processed using Stata 17 software. The analysis used chi-square statistics to determine the association between dependent variables and the various predictor variables at the bivariate level. Furthermore, multinomial logistic regression was used at the multivariate level to estimate the relative risk ratio (RRR) of experiencing physical or emotional violence or reporting both relative to experiencing none of these forms of IPV. The method is used when the outcome variable has two or more categories and was relevant in this case because the individual could have experienced physical or emotional violence, or both. The cut-off point for the significance level was 0.05. All variables were tested for multicollinearity to ensure that the variables did not interact with each other. Variables that had significant interaction with others were excluded from the analysis. They are the household wealth index that was correlated with education and place of residence and the number of children that was correlated with the number of years an individual has been in a relationship and the age of the respondent.

### Dependent variable

Physical violence was measured based on the response to the following questions^35^:

*Have you (a) ever been pushed, shaken or had something thrown by your husband/partner; (b) ever been slapped by your husband/partner; (c) ever been punched with a fist or hit by something harmful by your husband/partner; (d) ever been kicked or dragged by your husband/partner; (e) ever been strangled or burnt by your husband/partner; (f) ever been attacked with a knife, gun or other weapon by your husband/partner; (g) ever had your arm twisted or hair pulled by your husband/partner?*

Emotional violence, on the other hand, was based on the following questions^35^:

*Did your husband/partner ever (a) say or do something to humiliate you in front of others?; (b) threaten to hurt or harm you or someone you care about?; (c) insult you or make you feel bad about yourself?*

The outcome variable in the study was then created with four categories, coded ‘0’ when the respondent did not experience emotional or physical violence; ‘1’ to report physical, but not emotional violence; ‘2’ when the respondent experienced only emotional violence and ‘3’ when both emotional and physical were reported.

### Predictor variables

Independent variables were countries (Zimbabwe and Zambia); age group (15–24 years, 25–34 years, 35–44 years and 45–54 years); residence (rural and urban) and duration of the relationship (5 years or less, 6–10 years, 11–15 years and 16 or more years).

The other variables included the number of other wives (no other wife, 1 additional wife, 2 or more); education (no education, primary, secondary and higher); employment (not working, worked in the past year and is currently working); and if the respondent ever witnessed the father beating her mother (yes, no). Similarly, the partner’s employment status variable was as follows: not working, worked in the past year and is currently working; whether the partner drinks alcohol (yes or no). The acceptance of IPV was measured by asking whether the respondent believed that beating of the wife was justified under any of the following conditions: ‘arguing with partner/husband’; ‘going out without telling the partner/husband’; ‘burning food’; ‘refusing to have sex with the partner/husband’ and ‘neglecting the children’. The variable was coded as ‘1’ if the response was ‘yes’ for any of the questions and ‘0’ otherwise. Controlling behaviour was measured by asking if the partner does any of the following: ‘gets jealous if you talk to other men’, ‘accuses respondents of unfaithfulness’, ‘limiting the contact with the family’, ‘not allowing respondents to meet female friends’ and ‘insists on knowing where the partner is’. The variable was coded ‘0’ if the response was ‘no’ in all cases and ‘1’ if the response was positive for any of these questions.

The dummy variable for the use of alcohol by the partner and if the father ever beat his mother were coded ‘1’ if the condition existed and ‘0’ otherwise.

### Ethical considerations

Ethical approval to use the Survey Datasets was received from The Demographic and Health Surveys (DHS) Program. ICF International dated 17 September 2024. All procedures performed in studies involving human participants were in accordance with the ethical standards of the institutional and/or national research committee and with the 1964 Helsinki Declaration and its later amendments or comparable ethical standards. The study used secondary data from ICF International in partnership with the host countries, all standard ethical procedures were followed to gain access to data, in partnership with the host countries, Zambia and Zimbabwe. All ethical procedures were followed during data collection by the DHS to ensure respondents’ confidentiality, safety and dignity, and that they participated voluntarily. The DHS interviewers were trained to deal with sensitive topics such as domestic violence. The DHS protocol was approved by the Institutional Review Board after a thorough review to ensure compliance with the highest ethical standards. The following are some of the considerations during data collection as part of the standard DHS: only one woman per household was randomly selected for interview. Therefore, in households with more than one eligible woman, one eligible woman was selected using a CSPro random generation function. The interviewer had to make sure that no other person in the household besides the selected participant could know about the content of the interview. Informed consent was sought from the respondent at the beginning of the individual interview. Participants were informed that the questions could be sensitive and reassured of the confidentiality of their responses. Privacy was ensured for participants. The interviewers received specific training on how to collect data securely, confidentially, and ethically.

More information on the ethical standards of the DHS Programme can be found here: https://dhsprogram.com/Methodology/Protecting-the-Privacy-of-DHS-Survey-Respondents.cfm.^36^

## Results

Of the 11 778 respondents (see Table 1), 12.0% and 9.7% of the respondents reported experiencing physical and emotional violence alone, respectively, while the highest percentage (21.0%) suffered multiple IPV incidents (both physical and emotional). The majority of the respondents (54.0%) were from Zambia and the age group 25 years to 34 years contributed the most (39.0%) of the respondents. A higher percentage (61.0%) lived in rural areas, and almost two thirds (60.0%) had a relationship for 11 years to 15 years. More than half (57.0%) did not support wife-beating, meaning that 43.0% were in support. The largest support for wife-beating was in Zambia, with 45.0% supporting compared to 38.0% in Zimbabwe. While 61.0% indicated that they have a partner with controlling behaviour. Furthermore, 31.0% of their mothers had witnessed their father physically abused by their mother. Regarding the characteristics of the partners, more than half (54.0%) of the partners had a secondary level of education and 50.0% were working at the time of the survey. Furthermore, 38.0% of partners were using alcohol, while 68.0% had one of the controlling behaviours.

**TABLE 1:** Sample characteristics (weighted).

Main variable	Variables	Frequency (*n*)	%
Type of violence	None	6635	56.33
	Physical only	1443	12.25
	Emotional only	1144	9.72
	Both types of violence	2556	21.70
Country	Zambia	6439	54.66
	Zimbabwe	5340	45.34
Age group (years)	15–24	2632	22.35
	25–34	4618	39.20
	35–44	1971	16.74
	45–54	2557	21.71
Residence	Urban	4576	38.35
	Rural	8069	61.15
Duration (relationship)	5 years or less	2974	25.25
	6–10 years	2513	21.34
	11–15 years	2121	60.18
	16 years or more	4169	35.40
Number of other wives	No other wife	8501	87.45
	One additional wife	867	8.92
	Two or more additional wives	249	2.54
	Don’t know	106	1.10
Educational level	No education	745	6.33
	Primary	4853	41.20
	Secondary	5464	46.39
	Higher	718	6.08
Employment	Not working	4715	40.03
	Worked in the past year	1075	9.15
	Currently working	5986	50.82
Partner education	No education	341	3.51
	Primary	2871	29.54
	Secondary	5302	54.55
	Tertiary	943	9.71
	Don’t know	261	2.69
Partner’s employment status	Not working	1225	12.60
	Currently working	7524	77.41
	Worked past year	941	9.69
Accept wife-beating	No	6831	57.99
	Yes	4948	42.01
Controlling behaviour	No	3727	31.64
	Yes	8051	68.36
Partner drinks alcohol	No	7203	61.15
	Yes	4576	38.85
Father ever beat mother	No	7210	61.22
	Yes	3696	31.38

**Total**		**11 778**	**100**

Table 2 shows the relationship between IPV and the different socioeconomic variables. Slightly less than a quarter (23.31%) of the participants in Zambia reported experiencing more than one type of IPV compared to 19.0% of those in Zimbabwe. Similarly, Zambian women reported the highest number (13.38%) of those who experienced physical IPV, while the highest percentage in Zambia experienced emotional abuse (11.73%). Age and place of residence were not statistically significant (*p* > 0.05).

**TABLE 2:** Intimate partner violence prevalence by socioeconomic factors.

Variables	Never	Physical	Emotional	Both	Total	*p*
**Country**	-	-	-	-	-	< 0.001
Zimbabwe	57.610	10.98	11.73	19.77	6439	-
Zambia	55.270	13.38	8.08	23.31	5340	-
**Age group (years)**	-	-	-	-	-	0.100
15–24	58.200	12.99	9.03	19.76	2632	-
25–34	54.160	12.79	10.31	22.74	4618	-
35–44	57.670	10.08	10.28	21.97	1971	-
45–54	58.040	11.42	8.92	21.61	2557	-
**Residence**	-	-	-	-	-	0.180
Urban	55.810	11.66	10.07	21.83	4576	-
Rural	56.660	12.63	9.09	21.62	8069	-
**Educational**	-	-	-	-	-	< 0.001
No education	50.550	15.55	10.69	23.26	745	-
Primary	53.460	13.31	8.70	24.53	4853	-
Secondary	57.920	11.61	10.53	19.93	5464	-
Higher	69.660	6.56	9.34	14.43	718	-
**No. of additional wives**	-	-	-	-	-	< 0.001
0	59.860	12.34	9.26	18.54	8501	-
1	48.380	16.01	11.55	24.05	867	-
2 or more	46.270	13.15	16.97	23.61	249	-
Don’t know	51.160	11.26	11.65	26.00	106	-
**Relationship duration**	-	-	-	-	-	< 0.001
5 years or less	61.810	11.82	9.48	16.88	2974	-
6–10 years	52.290	13.25	10.46	24.00	2513	-
11–15 years	54.210	12.21	9.79	23.80	2121	-
16+ years	55.940	11.97	9.40	22.69	4169	-
**Employment status**	-	-	-	-	-	< 0.001
Not working	59.940	12.45	8.95	18.66	-	-
Worked in the past year	54.050	10.87	9.59	25.49	-	-
Currently working	53.900	12.35	10.34	23.41	-	-
**Partner employment status**	-	-	-	-	-	< 0.050
Not working	60.690	12.04	8.81	18.45	-	-
Currently working	58.825	12.46	9.58	19.14	-	-
Worked past 12 months	52.520	15.44	11.64	20.40	-	-
**Partner’s education**	-	-	-	-	-	< 0.001
No education	56.350	15.17	7.25	21.20	-	-
Primary	53.840	13.70	9.24	23.21	-	-
Secondary	59.390	12.51	10.12	17.97	-	-
Tertiary	67.700	10.55	10.52	11.23	-	-
**Accept wife-beating**	-	-	-	-	-	< 0.001
Yes	48.260	15.30	10.06	26.37	-	-
No	62.180	10.04	9.47	18.31	-	-
**Controlling behaviour**	-	-	-	-	-	< 0.001
No	80.690	8.43	4.61	6.27	3727	-
Yes	45.050	14.02	12.08	28.87	8051	-
**Partner drinks alcohol**	-	-	-	-	-	< 0.001
Yes	66.460	10.44	8.64	14.46	7203	-
No	40.390	15.10	11.40	33.10	4576	-
**Mother ever beaten**	-	-	-	-	-	< 0.001
No	63.100	10.23	9.19	17.51	7210	-
Yes	46.100	13.71	11.97	28.24	3696	-
Don’t know	52.600	14.20	11.20	22.00	872	-

Education was found to be a protective factor against IPV. Individuals with higher levels of education reported lower prevalence rates of IPV in almost all categories (*p* < 0.001). For example, only 14.5% of women with higher education experienced more than one IPV incident, compared to 23.0% of those without formal education. Furthermore, physical abuse decreased significantly with increasing educational levels, from 15.0% among those without education to 6.5% among those with higher educational attainment (*p* < 0.001). In contrast, employment status significantly affected the prevalence of IPV. The unemployed participants reported the lowest rates of multiple and physical IPV incidents. Specifically, the prevalence of both IPV was nearly five percentage points lower among unemployed women (18.6%) compared to those who worked (23.4%), with a statistically significant difference (*p* < 0.001).

In addition, polygamous relationships appeared to contribute to higher IPV rates. Women in polygamous relationships, where there were two or more additional wives, experienced an increase in multiple IPV incidents from 18.5% to 23.6% compared to those in monogamous relationships, also with a statistically significant difference (*p* < 0.001). Emotional IPV rose by seven percentage points, increasing from 9.26% to 16.97% under these conditions. The prevalence rate related to the number of years a woman has been in a relationship follows an inverted *U*-shape. It is lowest for those in relationships lasting 5 years or less, reaches its highest point for those in relationships of 6 years to 10 years, and then decreases again for longer relationships. Furthermore, the highest percentage of participants who supported physical abuse of women reported encountering both forms of abuse in the reporting period. The highest percentage (26.0%) reported multiple IPV incidents versus 18.0% of those who did not endorse such abuse.

The characteristics of a partner have been identified as critical factors in the prevalence of IPV. Women whose partners were employed in the past year reported the highest incidence of abuse, 20.0% experiencing multiple types of IPV, 15.0% facing physical violence, and 11.6% experiencing emotional abuse. In addition, higher levels of education among partners were associated with lower rates of IPV. For example, the prevalence of multiple IPV incidents was 11.0% among women with educated partners, compared to 21.0% among those with partners who had no formal education. Similarly, the rates of emotional and physical abuse were 6.0% versus 11.0% and 10.0% versus 15.0% for educated versus uneducated partners, respectively.

Moreover, more than a quarter (28%) of participants with partners who displayed any of the controlling behaviours were subjected to multiple IPV incidents compared to only 6% of those who did not have such partners. Similarly, having a partner who drinks alcohol increased multiple abuse incidents to 33% compared to 14% of women whose partners did not consume alcohol.

Women who approved IPV reported higher levels of physical and emotional violence. About 25% of those who endorsed such behaviour experienced violence, compared to only 18% of those who disapproved. In addition, the highest rates of all three types of violence were found among individuals whose partners exhibited controlling behaviour, with 28% experiencing both physical and emotional violence, compared to just 6% of those whose partners did not display such behaviour. Furthermore, individuals whose spouses consumed alcohol had the highest rates of experiencing all three types of violence. This was particularly evident among those who had suffered dual IPV, where the rate was more than double compared to those whose partners did not drink alcohol. Finally, people who witnessed their mother being assaulted by her partner reported the highest percentage (28%) of experiencing multiple forms of IPV, compared to only 17% of those who did not witness such events.

Table 3 presents the RRRs for reporting emotional and physical IPV, as well as cases where an individual reported both instances in comparison to individuals who did not experience any form of IPV. At the country level, while reporting physical and multiple instances of IPV was not statistically significant, respondents from Zambia were significantly more likely to have experienced emotional violence, with an RRR of 1.69 (95% confidence interval [CI]: 1.39–2.05). Age was not statistically significant for any of the different types of IPV. Among the urban dwellers, the risk of experiencing emotional IPV was reduced by 28% (RRR = 0.72, 95% CI: 0.59–0.88) relative to those in the rural areas.

**TABLE 3:** Relative risk ratio estimates for physical, emotional and multiple intimate partner violence.

Variable	Physical	95% CI	Emotional	95% CI	Both	95% CI
**Country**						
Zimbabwe	Ref	-	Ref	-	Ref	-
Zambia	0.88	0.74–1.06	1.69***	1.39–2.05	1.02	0.86–1.21
**Age group (years)**						
15–24	Ref	-	Ref	-	Ref	-
25–34	0.98	0.74–1.29	1.06	0.79–1.43	1.01	0.81–1.26
35–44	0.82	0.54–1.24	1.03	0.64–1.64	0.89	0.63–1.25
45–54	0.83	0.45–1.40	0.80	0.47–1.37	0.97	0.67–1.40
**Place of residence**						
Urban	0.89	0.73–1.08	0.72***	0.59–0.88	0.89	0.73–1.07
Rural	Ref	-	Ref	-	Ref	-
**Educational level**						
No education	2.54**	1.46–4.73	2.60***	1.39–4.87	1.24	0.71–2.16
Primary	2.08**	1.26–3.25	1.53	0.90–2.60	1.18	0.74–1.89
Secondary	1.88**	1.18–2.98	1.50	0.91–2.48	1.13	0.72–1.76
Higher	Ref	-	Ref	-	Ref	-
**Employment**						
Not working	Ref	-	Ref	-	Ref	-
Past year	0.84	0.64–1.11	1.00	0.72–1.38	1.10	0.83–1.45
Currently working	1.19*	1.01–1.41	1.28**	1.07–1.54	1.19*	1.00–1.40
**Duration (years)**						
0–5	Ref	-	Ref	-	Ref	-
6–10	1.40**	1.10–1.77	1.20	0.90–1.61	1.72***	1.39–2.13
11–15	1.37*	1.03–1.82	1.05	0.72–1.53	1.77***	1.38–2.27
16 or more	1.41	0.99–2.01	1.16	0.73– 1.85	1.73***	1.28–2.34
**Additional wives**						
0	Ref	-	Ref	-	Ref	-
1	1.52**	1.17–1.98	1.49*	1.07–2.07	1.38***	1.11–1.71
2 or more	1.71	0.90–3.24	2.60***	1.55–4.36	1.94*	1.10–3.39
Don’t know	1.16	0.58–2.33	1.57	0.39–6.30	1.86*	1.05–3.28
**Wife-beating justifiable**						
Yes	1.64***	1.39–1.92	1.25*	1.02–1.53	1.52***	1.31–1.78
No	Ref	-	Ref	-	Ref	-
**Controlling behaviour**						
Yes	2.43***	2.05–2.90	4.04***	3.19–5.12	5.85***	4.62–7.39
No	Ref	-	Ref	-	Ref	-
**Partner drinks alcohol**						
No	Ref	-	Ref	-	Ref	-
Yes	2.08***	1.79–2.42	1.91***	1.58–2.31	3.07***	2.62–3.61
**Father ever beat mother**						
No	Ref	-	Ref	-	Ref	-
Yes	1.78***	1.50–2.11	1.71***	1.42–2.07	2.07***	1.81–2.38
Don’t know	1.69***	1.30–2.19	1.38*	1.02–1.88	1.53***	1.20–1.95
**Partner’s age**	0.98	0.96–1.00	0.99	0.98–1.01	0.98*	0.97–0.99
**Employ status (Partner)**						
Worked on in the past year	1.47*	1.04–2.08	1.48	0.78–1.45	1.25	0.86–1.34
Worked in past 7 days	1.03	0.80–1.33	1.06	0.98–2.24	1.08	0.92–1.66
Not working	Ref	-	Ref	-	Ref	-
**Partner education**						
No education	0.87	0.54–1.41	0.65	0.34–1.24	1.55	0.90–2.65
Primary	0.93	0.64–1.35	0.98	0.67–1.43	1.96***	1.37–2.81
Secondary	0.85	0.60–1.20	0.86	0.60–1.23	1.43*	1.03–1.98
Tertiary	Ref	-	Ref	-	Ref	-
**Constant**	0.05***	0.07–0.47	0.01***	0.02–0.16	0.01***	0.05–0.24

CI, confidence interval.

*** , *p* < 0.005;

** , *p* < 0.01;

* , *p* < 0.05.

There were no significant differences between women with different levels of education for reporting multiple cases of abuse. However, women without formal and primary levels of education were more than twice more likely to experience physical abuse relative to those with tertiary education with risk ratios of 2.54 (95% CI: 1.46–4.73) and 2.08 (95% CI: 1.26–3.25), respectively. And the risk was higher by 88% (95% CI: 1.18–2.98) for those who attained a secondary education. Similarly, emotional abuse more than doubled (RRR = 2.60, 95% CI: 1.39–4.87) for women without an education. Multiple and physical IPV incidents were 1.19 times greater (95% CI: 1.00–1.40 and 95% CI: 1.01–1.41) among those working and rose by 1.28 times more (95% CI: 1.07–1.53) for emotional abuse.

The relative risk associated with the duration of a woman’s relationship exhibits an inverted *U*-shape. Compared to women who have been in a relationship for 5 years or less, the risk of experiencing both instances of abuse increased by 1.72 times (95% CI: 1.39–2.13) than those in relationships lasting 6 to 10 years. For women in relationships lasting 11 to 15 years, the risk increased further to 1.77 times (95% CI: 1.38–2.27). After 15 years, the risk decreased slightly but is still 73% higher (95% CI: 1.28–2.34) compared to those with 5 years or fewer in relationships. Furthermore, respondents in polygamous marriages were more likely to experience IPV compared to those in monogamous relationships. The risk of multiple IPV incidents was 38% higher (95% CI: 1.11–1.71) when the respondent had a co-wife and increased to 94% (95% CI: 1.10–3.39) with two or more co-wives. Emotional IPV increased almost threefold (RRR = 2.60, 95% CI: 1.55–4.36) when there were two or more co-wives. Furthermore, supporting wife-beating significantly predicted whether a person would experience IPV. The relative risk of reporting both cases increased by 52% (95% CI: 1.30–1.78) for women who endorsed wife beating. The risk of physical violence in this case was highest (RRR = 1.64, 95% CI: 1.39–1.92) followed by emotional abuse (RRR = 1.25, 95% CI: 1.02–1.54). Furthermore, the risk of reporting both cases more than doubled (RRR = 2.07, 95% CI: 1.82–2.38) for a woman who witnessed her mother being assaulted by her father.

The results indicate that partner characteristics significantly influence the prevalence of IPV in different ways. Participants whose partners drank alcohol were three times more likely (RRR = 3.06, 95% CI: 2.61–3.59) to have reported experiencing multiple cases of abuse. The risk of physical and emotional abuse alone was more than two times (RRR = 2.07, 95% CI: 1.78–2.41 and RRR = 1.91, 95% CI: 1.58–2.31), respectively. In addition, having a partner with controlling behaviour was the greatest risk factor compared to other factors in the study. The risk of reporting more than one case of IPV was nearly six times (RRR = 5.88, 95% CI: 4.65–7.43) for women with controlling partners. The risk of emotional abuse was four times higher (RRR = 4.07, 95% CI: 3.21–5.16) while physical abuse increased 2.45 times more (95% CI: 2.06–2.91) compared to women who did not have authoritative partners.

Partner education was found to have a protective effect on women. Compared to participants with partners with tertiary education, those whose partners had completed only secondary and primary educational levels were 1.43 times more likely (95% CI: 1.03–1.98) and almost twice more likely (RRR = 1.96, 95% CI: 1.37–2.81) to report both instances of IPV. However, there were no statistically significant differences in emotional and physical IPV (*p* > 0.05). Furthermore, each additional year of the partner’s age was associated with a 2% decrease in the probability of reporting multiple IPV (95% CI: 0.97–0.99). Lastly, the employment status of a partner was statistically significant only for physical IPV, and women whose partners worked in the past year were 1.4 times more likely (95% CI: 1.03–2.07) to report physical violence compared to those whose partners did not work.

## Discussion

Previous studies using data from DHS have examined the extent of various forms of IPV separately.^1,2,11,12,13^ This study improves existing research by providing a detailed assessment of the prevalence and predictors of physical and emotional violence and cases where individuals report experiencing physical and emotional abuse simultaneously. The study revealed that 44.0% of participants reported suffering from either physical, emotional or both forms of IPV. Notably, nearly a quarter (21.7%) reported experiencing multiple instances of IPV, while 12.0% indicated they had only suffered physical and 9.7% emotional abuses, respectively.

The effects of different predictors in the study vary with each type of violence. Age was not statistically significant, contradicting findings from the previous study. However, the variable became insignificant when the partner’s age was included in the analysis (results not shown).^11^ This is because the age of a woman is positively associated with the age of her partner. On the contrary, respondents in advanced age categories usually have partners of similar ages. The findings corroborated previous studies that found that women in the workforce face a higher risk of IPV.^37^ Furthermore, this study found that physical violence is also high when the partner is employed. Authors from Tanzania indicated that employed women often spend more time away from home, which can lead to jealousy of their partners and result in IPV.^38^ In addition, interactions with individuals of the opposite sex in the workplace may create insecurity in the woman’s partner. The partner may want to control how the woman’s earnings are used while withholding his money. Such dynamics can lead to difficulties in balancing family and work demands, creating additional tensions within the family.^39^

Regarding the time spent in a relationship, the risk of IPV in this study followed an inverted *U*-shape pattern, peaking in relationships that have lasted between 6 years and 10 years for physical IPV and beyond 10 years for reporting both physical and emotional IPV, respectively.^2,9,31,32^ Women who experience abuse can spend the initial years trying to resolve issues within the relationship. However, they often choose to leave when these problems continue over time. This could explain the lower prevalence of abuse among women in longer relationships.^5,6^ As time passes, couples may develop a better understanding of each other, which can lead to increased respect and trust. This improvement in your relationship can decrease the likelihood of partner violence. The results showed that the risk of experiencing IPV was higher for each additional co-wife a woman had. The greatest risks were incidents of emotional and multiple IPV, respectively. Being married to one man can create competition for the partner’s attention, leading some women to feel emotionally neglected or abused.^6^

Furthermore, the increased demand for resources associated with each additional wife and their children can create tension within the family, especially when resources are limited, potentially resulting in IPV.^40,41,42^ This implies that the family is likely to experience financial constraints resulting in family conflicts. For instance, when the wife feels not supported by the husband because of the limited or split resources because of the new wife (polygamy). A previous study found that women who saw their mothers being abused by their fathers were more likely to experience violence themselves,^5,9^ which aligns with the findings of this study. Some women may look for partners who resemble their fathers in different ways, including masculinity characteristics that are associated with violent behaviour in some cases.

Women who believe that IPV is justifiable are at the highest risk of experiencing it themselves. This risk is particularly significant for both physical violence and multiple forms of IPV. However, this belief may serve as a coping mechanism in societies where partner violence is common. One study found that victims of IPV have fewer mental problems when they endorse this behaviour.^23^ Patriarchal values can lead to the normalisation of abuse against women, as societal pressure may make such behaviour seem acceptable. Women who accept these norms are often less likely to report incidents of abuse to authorities, which diminishes the accountability of perpetrators and may encourage them to commit further acts of violence.

The study found that the spousal characteristics significantly influenced the prevalence of IPV in various ways. Factors linked to partners that increased the risk of IPV included controlling behaviour, alcohol consumption, recent employment and lower educational attainment. In contrast, a partner’s age was found to serve as a protective factor against IPV. As noticed in another study,^3^ the risk of IPV was significantly higher when a partner showed controlling or authoritative behaviour, increasing the risk six times after adjusting for other variables. Furthermore, the findings confirmed that the risk of IPV was elevated among those whose partners consumed alcohol, in accordance with results from other studies.^31^

### Strengths and limitations of the study

This study took advantage of the latest data available in the two countries and therefore provides up-to-date information. Differences in countries might be influenced by the time difference between data collections, as trends in IPV change over time. The study used cross-sectional data and therefore it is not possible to infer causality between the variables. It is recommended that future research in this area explores the sequencing of the different IPV to establish how they affect each other.

### Recommendations

Education for both men and women, particularly at secondary and tertiary levels is critical to reducing IPV,^1^ and therefore, we recommend that policymakers improve education generally in society. Efforts should focus on empowering women to oppose any form of IPV and to reject justifications for violence against partners. This could involve campaigns aimed at empowering women in different ways, including addressing gender imbalances and encouraging women to report violence to the authorities, rather than accepting it as a normal part of life. Intimate partner violence perception as a normal behaviour needs to change, this has to do with cultural norms and attitudes towards women and abusive men. Zimbabwe and Zambia need to ensure that all cases of IPV are recorded and per arrest. The study’s results indicate that there is a need for evidence-based interventions against IPV, which are diverse and span individual, community, healthcare and policy levels. These interventions have been studied in various contexts in Africa and are supported by research from organisations such as the WHO, Centers for Disease Control and Prevention (CDC), and academic institutions.^25^ Here’s a breakdown of effective interventions with supporting evidence:

- Healthcare-based interventions provide training to healthcare providers to screen patients (especially women) for IPV and offer brief counselling, safety planning and referrals.^27^
- Trauma-informed care ensures that care providers are trained to recognise and sensitively respond to trauma.^28^
- Community and educational interventions, school-based relationship education teach adolescents about healthy relationships, gender norms, and communication.^29^
- Community mobilisation: engaging communities to challenge norms that support IPV and promote bystander intervention.^28^
- Legal and policy interventions: strengthening legal protections, enacting and enforcing laws that criminalise IPV and protect survivors.^1^
- Mandatory arrest policies and protection order: protection orders can be effective if enforced, but mandatory arrest laws may deter reporting in some cases.^1^
- Economic empowerment interventions: microfinance and gender training which combines small loans and/or savings with training on gender equality and IPV.^16^
- Cash transfers: conditional and unconditional cash transfers in Latin America and Africa have shown reductions in IPV, possibly by reducing economic stress and improving bargaining power.^43^
- Perpetrator intervention programmes: Batterer Intervention Programs (BIPs), group-based cognitive behavioural therapy (CBT) aimed at changing abusive behaviour.^44^
- Technology-based interventions, such as, Safety Apps and Online Resources, for instance, myPlan app help users assess relationship safety and create safety plans.^45^

It is recommended that these interventions should have key principles for effective interventions:^46,47^ multisectoral coordination (health, legal, education, social services), cultural adaptation of interventions, survivor-centred and trauma-informed approaches, and sustained community engagement and policy support.

## Conclusion

There are several critical factors influencing the risk of IPV, such as partner characteristics. The most significant risk factors for dual IPV were partners exhibiting controlling behaviour and those who consumed alcohol. Having a controlling partner increased the risk of reporting more than one IPV incident by nearly six times, and partners’ alcohol consumption more than tripled the risk of multiple abuse cases. Partner’s employment status was also linked to physical IPV, with women whose partners worked in the past year being more likely to report physical violence. Relationship dynamics and polygamous relationships significantly increased the risk of IPV, with the risk of multiple IPV incidents being higher with one co-wife and higher with two or more co-wives. Emotional IPV almost tripled with two or more co-wives. The duration of a relationship showed an inverted *U*-shape pattern for IPV risk, with the highest probability of experiencing both forms of IPV occurring in relationships lasting between 6 years and 15 years. Individual and societal factors women who themselves supported the justification of wife-beating were at a significantly higher risk of experiencing IPV, with the relative risk of reporting both forms increasing. Conversely, educational attainment emerged as a crucial protective factor. Higher education levels for both women and their partners were consistently associated with a decrease in IPV prevalence.

This study’s contribution lies in its detailed assessment of the prevalence and predictors of both physical and emotional violence, particularly focusing on cases where individuals experience both simultaneously, an area previously underexplored. By using comprehensive DHS data from Zambia and Zimbabwe, the study provides up-to-date, context-specific insights that are valuable for shaping policies and programmes to prevent gender-based violence. The emphasis on mental healthcare for women who experience IPV is also a notable contribution. Based on these findings, a multi-faceted approach is recommended to address IPV effectively, for instance, coordinated responses, context-specific strategies, support systems, and education and empowerment. Addressing the widespread issue of dual IPV in SSA requires comprehensive interventions that tackle individual, relational and societal risk factors, while simultaneously bolstering protective measures such as education and support systems
